# Supercritical Fluid Chromatography with Photodiode Array Detection in the Determination of Fat-Soluble Vitamins in Hemp Seed Oil and Waste Fish Oil

**DOI:** 10.3390/molecules23051131

**Published:** 2018-05-10

**Authors:** Katarzyna Tyśkiewicz, Roman Gieysztor, Izabela Maziarczyk, Paweł Hodurek, Edward Rój, Krystyna Skalicka-Woźniak

**Affiliations:** 1 New Chemical Syntheses Institute, Supercritical Extraction Department, Puławy 24-110, Poland; roman.gieysztor@ins.pulawy.pl (R.G.); izabela.nakonieczna@ins.pulawy.pl (I.M.); edward.roj@ins.pulawy.pl (E.R.); 2 Medical University of Wrocław, Faculty of Health Sciences, Department of Angiology, Wrocław 50-367, Poland; pawel.hodurek@hotmail.com; 3 Department of Pharmacognosy with Medicinal Plant Unit, Medical University of Lublin, Lublin 20-093, Poland; kskalicka@pharmacognosy.org

**Keywords:** fat-soluble vitamins, hemp seed oil, purification, supercritical fluid chromatography, waste fish oil

## Abstract

In the presented study for the first time a new, optimized, fast SFC (supercritical fluid chromatography) method was applied to separate in one run fat-soluble vitamins from waste fish oil, including *cis*-and *tran*s-retinyl palmitate, *cis*- and *trans*-retinyl acetate, retinol, α-tocopherol, β-tocopherol, γ‑tocopherol, δ-tocopherol, ergocalciferol (D_2_), cholecalciferol (D_3_), *cis*- and *trans*-phylloquinone (K_1_) and menaquinone-4 (K_2_-MK4). Vitamins were baseline separated on an Acquity UPC^2^ (ultra performance convergence chromatography) HSS C18 SB (highly strength chemically modified silica) column within 13 min. The influence of the stationary phase, such as Torus 1-AA (1-aminoanthracene), Torus Diol (high density diol), Torus DEA (diethylamine), BEH (silica with no bonding), BEH-2EP (2-ethylpirydine), CSH Fluoro-Phenyl (silica with fluoro-phenyl groups), column temperature, flow rate and back pressure on the separation of the compounds was described. The application of the modified saponification procedure allowed us to increase concentration in the sample prepared for the analysis of γ‑tocopherol from less than 1% (wt %) to 14% for the first time. In addition, α‑tocopherol, γ‑tocopherol, δ‑tocopherol and retinol were identified in waste fish oil. Vitamin purification and analysis in waste fish oil are reported for the first time here. Due to the short time and effectiveness of the proposed method, it can be easily applied in industrial processes.

## 1. Introduction

The main area of the interest for hemp seed oil concerns its beneficial properties influencing health and nutrition [1]. For instance, it affects the level of cholesterol, as well as blood pressure [2]. Moreover, due to the presence of antioxidant compounds, the risk of cardiovascular diseases and cancer may be decreased [3]. Taking into consideration the chemical composition of the hemp seed oil, it is characterized by a high content of linoleic acid (n-6) and linolenic acid (n-3) in the amounts of 50–70% and 15–25%, respectively [4,5]. According to Parker et al. [6], the content of fatty acids in cold-pressed seed oil may be up to 79 g per 100 g of the fatty acids. Another group of compounds present in the hemp seed oil are tocopherols, including all four isomers.

The vitamin content in fish oil may vary depending on the species as well as the part of the fish. According to Stancheva [7], the fish oil constituted retinol and α-tocopherol in the amount of 9–142 μg and 308–1113 μg per 100 g of the raw tissue, respectively, in four different fish species. However, the literature lacks information on the content of fat-soluble vitamins in waste fish oil.

The analysis of vitamins is a challenging task in many ways. Firstly, the vitamins are sensitive to heat, light, oxygen and other factors causing their isomerization. Moreover, unstable polyene chains in the vitamin structures increase their degradation [8,9,10]. On the other hand, fat-soluble and water-soluble vitamins constitute a group of compounds with different chemical structures and characteristics, which may be used while performing separation [11]. Another aspect is that SFC methods are performed at subcritical conditions, but also with the addition of co-solvent and low temperatures. Often, the mobile phase is intentionally kept at subcritical conditions, in which the properties of the mobile phase are continuous and satisfactory separation is obtained [12,13].

Various separation techniques have been applied in the analysis of vitamins in foods, vegetables, and biological samples. It was proved by a number of researchers that SFC is as effective as other analytical techniques in vitamin separation. In analytical studies by Galuba [14], the content of tocopherols (α-, γ-, δ-) analyzed by the HPLC (high performance liquid chromatography) and SFC (supercritical fluid chromatography) method was comparable. An increased number of applications in the pharmaceutical industry concerns the separation of isomers by SFC [15]. In previous studies, supercritical fluid chromatography equipped with different detectors was applied to separate tocopherols and tocotrienols [16,17,18], vitamin A and carotenoids [9,10,19,20], vitamin D [21,22] and vitamin K [15,23], as well as the mixture of fat-soluble vitamins [24,25] and water-soluble vitamins [12]. Thus far, there has been a lack of an efficient and fast methods for the one-step separation of a higher number of vitamins.

The aim of this study is to propose a new efficient method for the separation of fourteen fat-soluble vitamins with the use of supercritical fluid chromatography. The optimization includes the influence of different parameters, such as back pressure, temperature, flow rate and stationary phase on the separation of analytes. Moreover, the group of mentioned vitamins are to be purified and quantitated in hemp seed oil, and in the case of waste fish oil are to be identified and purified for the first time. 

## 2. Materials and Methods

### 2.1. Standards and Materials

Authentic analytical standards, retinol, retinyl palmitate (*cis*- and *trans*-isomer), retinyl acetate (*cis*- and *trans*-isomer), α-tocopherol, β-tocopherol, γ-tocopherol, δ-tocopherol, *cis*-phylloquinone (*cis*-K_1_), *trans*-phylloquinone (*trans*-K_1_), menaquinone-4 (K_2_-MK4), cholecalciferol (D_3_) and ergocalciferol (D_2_) were purchased from Sigma-Aldrich (Poznań, Poland). Vitamin D compounds and β-tocopherol were received from Sigma-Aldrich as standard solutions, 1 mg/mL in ethanol and 50 mg/mL in hexane, respectively. According to standard certificates, retinyl acetate as well as retinyl palmitate and phylloquinone (K_1_) contain 5% of *cis*-isomer. As for the materials for separation and analyses, commercially-available, cold-pressed hemp seed oil was used as well as the waste fish oil delivered by the A-SENSE company (Puławy, Poland). Each standard solution was injected alone to measure the corresponding retention time and UV spectrum (Sigma-Aldrich, Poznań, Poland).

### 2.2. Quantification

Individual stock solutions of authentic standards of retinol, retinyl palmitate, retinyl acetate, α‑tocopherol, γ-tocopherol, δ-tocopherol, *cis*-phylloquinone (*cis*-K1), *trans*-phylloquinone (*trans*-K1), menaquinone-4 (K2-MK4) were prepared in methyl tert-butyl ether (MTBE). The amount of 20 µL of β-tocopherol was diluted in 980 µL TBME (methyl tert-butyl ether) to obtain the concentration of 1 mg/mL. Due to the complexity of the studied matrices, the standard addition quantification method was applied in order to quantitate fat-soluble vitamins in hemp seed oil and waste fish oil before and after the saponification procedure. In this case, standard curves were obtained from five points by plotting the peak areas versus the volume of added standard to the sample. The response of the particular analyte was measured before and after the addition of the standard in three replicate. The method of standard addition quantification is precisely described by Steliopoulos [26]. The concentrations of unknown analytes were calculated according to the following Equation (1):

(1) $$\frac{V_{x}\times C_{s}}{V_{s}},$$

in which:

C_s_—the concentration of added standard
V_s_—the volume of sample analyzed
V_x_—the volume equivalent of standard measured by extrapolation on the x-axis

### 2.3. Instruments and Apparatus

The separation of fat-soluble vitamins was conducted on Waters Acquity Ultra-Performance Convergence Chromatography (UPC^2^) system (Waters, Milford, MA, USA) equipped with PDA (photodiode array) detection and a back pressure regulator working in the range of 10.5 MPa to 21.0 MPa. For data analysis, the Empower 3 software (3.0, Waters Corporation, Milford, MA, USA) was used. Seven 100 mm-length chromatographic columns with corresponding stationary phases purchased from Waters (Milford, MA, USA) were applied including HSS C18 SB (high strength chemically modified silica), Torus 1-AA (1‑aminoanthracene), Torus Diol (high density diol), Torus DEA (diethylamine), BEH (silica with no bonding), BEH-2EP (2-ethylpirydine) and CSH Fluoro-Phenyl. All of them had an internal diameter of 3.0 mm and different particle size (1.8 μm for silica bonded with C18 and 1.7 μm for the remaining stationary phases). Carbon dioxide (99.998% purity), which was used as the mobile phase in SFC, was purchased from Air Liquide. The HPLC grade methanol that was used as the second component of the mobile phase was purchased from Baker (purchased from Witko, Łódź, Poland). The compensation wavelength was set at 500–600 nm throughout the analyses. The wavelength for vitamin A was set to 320 nm and for the rest of the vitamins to 220 nm. Different conditions, such as stationary phase chemistry, back pressure, column temperature as well as flow rate were evaluated in order to obtain the best separation and optimized analysis time. The injection volume was 1 μL.

### 2.4. Partial Method Validation

In the case of the validation parameters of the method, the standard addition calibration curves obtained during the analysis proved to have appropriate linearity with correlation coefficients of 0.996 for menaquinone-4 (K_2_-MK4) and 0.994/0.996 for γ-tocopherol for hemp seed oil as well as 0.993 for α-tocopherol, 0.997 for γ-tocopherol, 0.996/0.993 for δ‑tocopherol and 0.996/0.994 for retinol for waste fish oil. Table 1 summarizes the typical standard addition calibration data (peak areas, slope, intercept) for hemp seed oil and waste fish oil samples before and after the applied saponification procedures. The slope-intercept values were obtained from the linear equation of x- and y-unknowns, in which x- is the concentration of added standard and y- is the signal of the analyzed sample. The linear equation is as follows (2):

(2) y=ax+b,

in which: *a* is the slope and *b* is the intercept.

Moreover, external standard calibration was performed in order to evaluate the accuracy of the provided method for the separation of fat-soluble vitamins. The *cis*-forms of the compounds were omitted. The coefficient of determination (R^2^) for all calibration curves was higher than 0.997 for all the analytes. The limits of detection (LOD) ranged between 0.10–1.17 µg/mL, whereas the limits of quantification were between 0.50–2.39 µg/mL (Table 2). Repeatability and intermediate precision were determined for the mixture of all fourteen vitamins at three different concentrations. The repeatability was measured by injecting the mixture on the same day in three replicates, whereas the intermediate precision was measured on the consecutive three days. The RSD (relative standard deviation) values were calculated according to the equation: RSD% = (standard deviation of the peak area/mean) × 100, and show that both the repeatability (RSD% < 3) and intermediate precision (RSD% < 6) were acceptable (Table 3). The accuracy of the method was conducted by spiking working standards into the sample of hemp seed oil at three different concentrations of each of the vitamins in the amount of 100 µg–300 µg for vitamins without the *cis*- isomers and 95 µg–285 µg for the retinyl acetate, retinyl palmitate and phylloquinone (K_1_). The sample recovery was presented as the percentage against the spiked amounts of each standard. The results presented in Table 3 indicate the suitability of the saponification method as the recovery for all fat-soluble vitamins was above 85%.

### 2.5. Vitamin Purification

Hemp seed oil (cold pressed) and waste fish oil tocopherols (α-tocopherol, γ‑tocopherol, δ-tocopherol) retinol and menaquinone-4 (K_2_-MK4) were purified using the modified saponification method of O’keefer and Ackman (1986), which was developed for vitamin purification in fish oil [27]. Briefly, about 2 g of hemp oil and waste fish oil were weighted separately into centrifuge tube and 30 mL of 90% ethanol solution was added. In the next step, 5 g of KOH, 40 mg of NaOH was dissolved in 5 mL and 10 mL of water, respectively. Ascorbic acid (1.7 g) was dissolved in a prepared NaOH solution. Then, 1.5 mL of KOH, 1 mL of ascorbic acid solution as well as edetic acid disodium salt were added to the oils. The tubes were placed in a water bath set to 75 °C for 15 min. The solutions were moved to separatory funnels and 150 mL of diethyl ether and 50 mL of water were added in order to obtain two phases. Ether phases were collected and washed two times with 50 mL water and dried with sodium sulfate. The solvent was evaporated at 34 °C. The residues were dried with nitrogen and TBME was used to bring the volume of 2 mL. The samples were filtered through 0.45 μm filter. No antioxidant was applied.

## 3. Results and Discussion

As far as we are aware, SFC was applied in the fat-soluble vitamin separation. However, a series of drawbacks were noticed. Rathi [25] applied UPC^2^ with photodiode array (PDA) detection to separate retinyl acetate, retinol, α-tocopherol, ergocalciferol (D_2_), cholecalciferol (D_3_), phylloquinone (K_1_), menaquinone-4 (K_2_-MK4), lycopene, lutein and β‑carotene. However, two stationary phases were evaluated, including Acquity UPC^2^ HSS C18 SB (100 × 3.0 mm; 1.8 μm) for carotenoids and Acquity UPC^2^ BEH (100 × 3.0 mm; 1.7 μm) for fat-soluble vitamins. Both Jumaah [13] and Yang [23] stressed the difficulty in separating the pairs of vitamin D_2_/D_3_ and K_1_/K_2_, as the difference in their structure lies in the number of double bonds in the case of vitamin K and the additional double bond and methyl group in the vitamin D_2_ structure in comparison to the vitamin D_3_ structure. 

In our study, for the first time we provide a new, efficient method for the one-step separation of fourteen compounds with satisfactory time and resolution between the compounds, including the mentioned pairs of vitamin D and vitamin K. The method is more valuable considering the fact that it provides the screening of all fat-soluble vitamins, which are in fact a complex mixture to separate. In addition, tocopherols may be separated simply by isocratic elution (data not shown). However, as it was studied in this paper, the mixture of fourteen compounds is more complicated. In the study, such parameters as stationary phase chemistry, back pressure, column temperature and the flow rate were tested.

### 3.1. Non-Polar Column Screening

#### 3.1.1. HSS C18 SB

**Figure 1:**
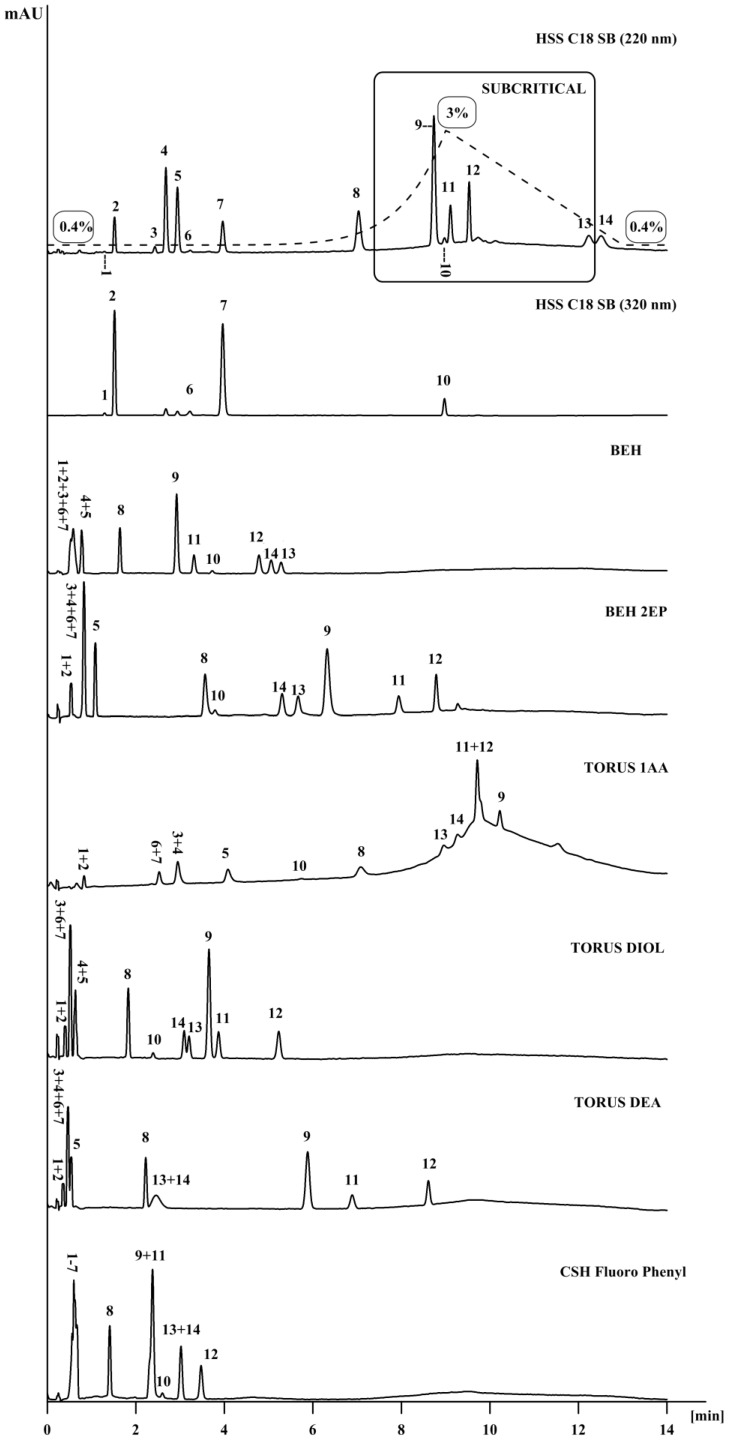
The separation of fat-soluble vitamins in different stationary phases. Mobile phase: CO_2_/methanol; gradient mode: initial 0.4% B, held for 1 min., increase to 3% B in 8 min., back to 0.4% B in 4 min.; flow rate: 2.3 mL/min; column temperature: 35 °C; ABPR (atmospheric back pressure regulator): 12.41 MPa; dash lines (---) show the gradient program, percentage of methanol in the mobile phase. (11-Z) *cis*-retinyl acetate (1), *trans*-retinyl acetate (2), *cis*-phylloquinone (*cis*-K_1_) (3), *trans*-phylloquinone (*trans*-K_1_) (4), menaquinone-4 (K_2_-MK4) (5) *cis*-retinyl palmitate (6), retinyl palmitate (7), α‑tocopherol (8), β-tocopherol (9), retinol (10), γ-tocopherol (11), δ‑tocopherol (12), ergocalciferol (D_2_) (13) and cholecalciferol (D_3_) (14).

Among seven stationary phases tested for the separation of fat-soluble vitamins, the Acquity UPC^2^ HSS C18 SB (100 mm × 3.0 I.D., 1.8 μm) column resulted in the separation of all fat-soluble vitamins (Figure 1). The retention depended upon the compound’s polarity (hydrophobic interactions are important). The separation was carried out at a column temperature of 35 °C with carbon dioxide and with methanol as the mobile phase, with the flow rate of 2.3 mL/min and the ABPR (atmospheric back pressure regulator) set to 12.41 MPa. The isocratic elution was conducted with 0.4% MeOH at the starting point and held for 1 min. Then, this was increased to 3% MeOH in 8 min (with a concave gradient curve = 10) and back to 0.4% MeOH in 4 min. The resolution (Rs) between compounds was >1, except that between cholecalciferol (D_3_) and ergocalciferol (D_2_), which was 0.96, although this did not disturb the quantitative analysis of the vitamins. The first to elute was retinyl acetate followed by *cis*-phylloquinone (*cis*-K_1_), *trans*-phylloquinone (*trans*-K_1_), menaquinone-4 (K_2_-MK4), retinyl palmitate, α‑tocopherol, retinol, β-tocopherol, γ‑tocopherol, δ tocopherol, cholecalciferol (D_3_) and ergocalciferol (D_2_).

#### 3.1.2. Torus 1-AA

The retention on the Torus 1-AA column was based on π–π interactions between the anthracene group of the stationary phase and aromatic groups of the analytes. Moreover, the *cis*- and *trans*-isomers of retinyl acetate, retinyl palmitate as well as phylloquinone (K1) were not separated and co-eluted on Torus-1AA column. As was found in this paper, in the case of fat-soluble vitamins, the column is not selective toward *cis*- and *trans*- isomers. In spite the fact that some compounds were not retained or co-eluted using the Torus 1-AA column, this column provided a good retention and chromatographic separation of ergocalciferol (D_2_) and cholecalciferol (D_3_). The hydrogen bonding mechanism also plays an important role in separating fat-soluble vitamins. The first to elute were compounds with no hydrogen bonding, whereas the last were tocopherols (β-, γ- and δ-). In the case of δ-tocopherol, the interaction of the aromatic group and the stationary phase was stronger than in the β- and γ-isomers, as it had only one hydrogen and one methyl group in the chroman ring.

### 3.2. Polar Column Screening

Both of the columns mentioned above have a non-polar nature and it is obvious for them to interact with the non-polar nature of vitamins. Taking into consideration the polar stationary phases, the interactions with vitamins are much different and in some cases, as in BEH-2EP, the separation of a critical pair (D_2_/D_3_) provided a satisfactory and improved resolution, as compared with HSS C18 SB. The same interactions were investigated by Jumaah [10] on the example of carotenoids. The non-polar stationary phases (HSS C18 SB and Torus 1-AA) were the best for the separation of the mentioned compounds. However, the polar stationary phases provided different interactions with the vitamins as well as different elution orders.

#### 3.2.1. BEH

In the case of the BEH column, phylloquinone (K_1_) and menaquinone-4 (K_2_-MK4), but also retinyl acetate and retinyl palmitate were not retained in this stationary phase. Due to the polar character of silica gel, non-polar compounds tend to elute before more polar compounds. The first to elute was α-tocopherol, which has one hydroxyl group and three methyl groups in the chroman ring. The remaining tocopherols eluted according to their increasing polarity. In the case of β-tocopherol and γ-tocopherol, which have the same molecular masses, the elution was dependent on the position of two methyl groups in the chroman ring. The last to elute was ergocalciferol (D_2_), which is the most polar of all the fat-soluble vitamins, as it has the most polar interactions with the stationary phase among other compounds. 

#### 3.2.2. BEH-2EP vs TORUS Diol

Similar to the BEH column, no retention was observed for the vitamin K compounds and ester forms of vitamin A on the columns with 2-ethylpirydine and diol groups bonded to silica under the same conditions as were employed on the HSS C18 SB column. Phylloquinone (K_1_), menaquinone-4 (K_2_-MK4) as well as retinyl acetate and retinyl palmitate were not retained on the diol column due to the lack of hydroxyl groups in their structures. The elution order for the rest of the compounds were the same on both columns. However, BEH-2EP provided better resolution between compounds due to stronger π–π interactions between the double bonds of the analytes and the double bonds of the stationary phase. This is seen well in the case of vitamin D compounds. BEH-2EP provided the best retention and selectivity of these compounds among the rest of the stationary phases. Ergocalciferol (D_2_) was retained longer, as it has an additional methyl group and a double bond in its structure, as compared to cholecalciferol (D_3_).

#### 3.2.3. Torus DEA

Vitamin K and ester forms of vitamin A were not retained on the Torus DEA column. On the other hand, the DEA column provided good retention and selectivity for separating tocopherols. The separation resulted in longer retention times for β-tocopherol, γ-tocopherol and δ-tocopherol due to more polar interactions as compared to the Diol column. However, retinol as well as cholecalciferol (D_3_) and ergocalciferol (D_2_) were not separated and co-eluted.

#### 3.2.4. CSH Fluoro-Phenyl

The retention on the CSH Fluoro Phenyl column is based on π–π interactions between the phenyl group of the stationary phase and unsaturated bonds of the analytes. However, as it was studied by Jumaah [13], the CSH column exhibits strong polar interactions with the –OH groups of the compounds. Vitamin K as well as retinyl acetate and retinyl palmitate were not retained as they do not possess a hydroxyl group. The interactions of the stationary phase with analytes are well seen in the case of vitamin D compounds, but also in the case of β‑tocopherol and γ-tocopherol. Both β-tocopherol and γ-tocopherol co-eluted as they differ only in the position of the methyl groups in the chroman ring, and the interactions of the stationary phase are the same for both compounds. The same example is true for vitamin D compounds. The last to elute was δ-tocopherol, as it has only one hydroxyl and one methyl group in the aromatic ring. 

### 3.3. Influence of Back Pressure

**Figure 2:**
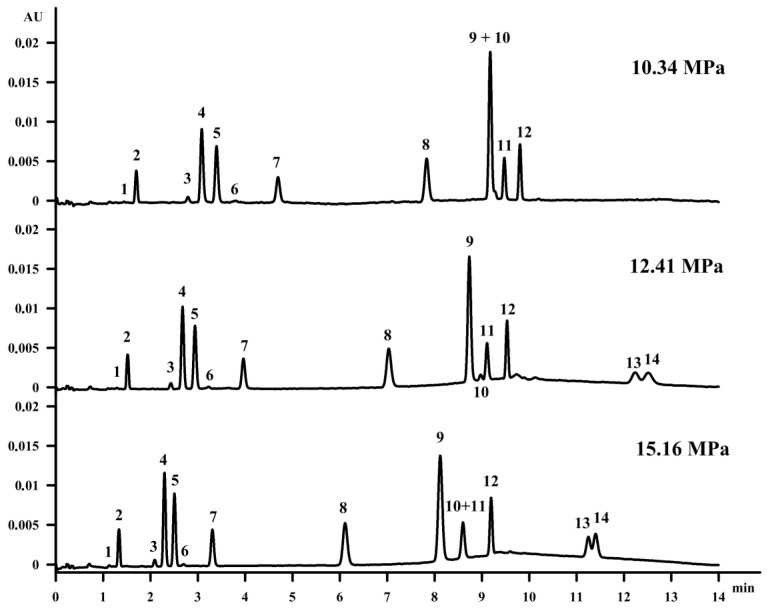
The influence of the back pressure on the separation of fat-soluble vitamins on the Acquity UPC^2^ HSS C18 SB column. The elution order is as in Figure 1.

Firstly, the influence of the back pressure on the vitamin separation was studied. Increasing the pressure at a constant temperature results in an increase of the density as well as of the mobile phase viscosity. However, capacity factor decreases that may be caused by the fact that compounds may be more soluble in the mobile phase when their density is increased [28]. Moreover, reaching a higher density of the mobile phase strengthens the solvent strength for the supercritical fluid [29]. Changing the pressure from 12.41 MPa to 10.34 MPa with a constant column temperature of 35 °C resulted in the co-elution of β‑tocopherol (peak 9) and retinol (peak 10), as well as the extension of vitamin D compound retention. On the other hand, it is well known in the field of SFC that when using a modifier with a mobile phase, the increase of the pressure does not improve the separation, which is proved in Figure 2.

### 3.4. Influence of Column Temperature

**Figure 3:**
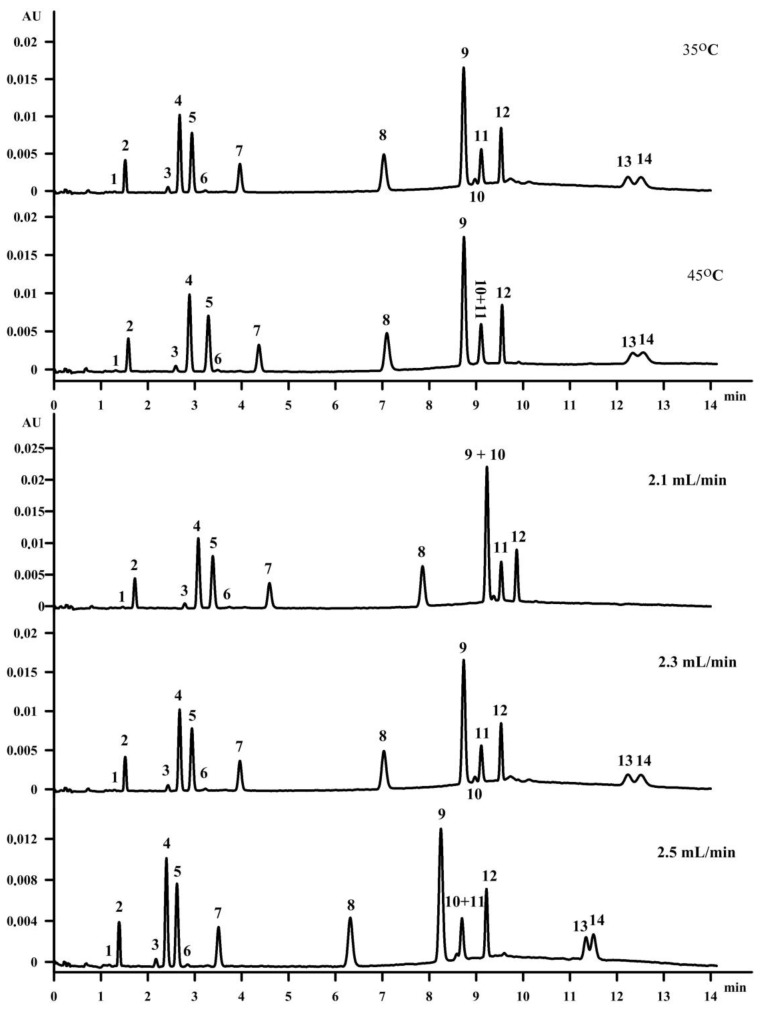
The influence of column temperature and flow rate on the separation of fat-soluble vitamins on an Acquity UPC^2^ HSS C18 SB column. The elution order is as in Figure 1.

When the temperature is increased at a constant pressure, changes in the solvent strength are observed. The retention depends on the temperature mainly due to the thermodynamic parameters of sorption. Changing the column temperature also has an influence on the diffusion coefficients, which increase with increased temperature [28]. The temperature of the column was studied in the range 35 °C−45 °C. If a number of compounds is separated during one analysis, usually increasing and decreasing the temperature can increase the resolution for one set of peaks and lower the resolution for the second set of peaks. This is shown in Figure 3. The temperature of 45 °C increased the resolution between the vitamin K isomers, but caused the co-elution of retinol (peak 10) and γ‑tocopherol (peak 11). Changing the column temperature from 45 °C to 35 °C increased the resolution between cholecalciferol (D_3_) (peak 13) and ergocalciferol (D_2_) (peak 14), as well as the vitamin K isomers to accepted values. Moreover, retinol (peak 10) and γ‑tocopherol (peak 11) were separated at the temperature of 35 °C. The separation of fat-soluble vitamins was also studied with a column temperature of 30 °C and 40 °C (data not shown). However, in both cases the separation on all fourteen compounds was not achieved.

### 3.5. Influence of Flow Rate

**Figure 4:**
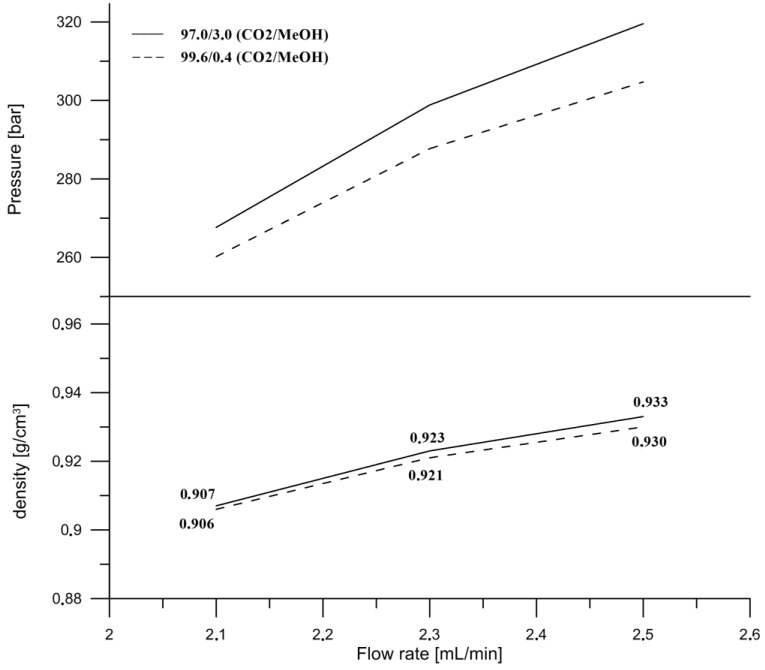
Inlet pressure and density of the mobile phase vs flow rate on the HSS C18 SB (high strength chemically modified silica) column at two different compositions of the mobile phase. ABPR was set at 12.41 MPa and column temperature at 35 °C.

The flow rate was the next parameter tested in this study. The influence of the flow rate on the separation in SFC was based on increasing the pressure drop when the flow rate increased, as it was presented in Figure 3. Consequently, with a higher pressure, the mobile phase density also increases [30]. The inlet pressures were measured during the isocratic elution for two mobile phase compositions at the initial conditions (99.6/0.4, *v*/*v*, CO_2_/MeOH) and maximum co-solvent (97.0/3.0, *v*/*v*, CO_2_/MeOH) at three different flow rates. The densities were calculated according to the composition of the mobile phase and the densities of the pure solvents at the particular conditions of the temperature of 35 °C and the pressures obtained at different flow rates. It might be concluded that a change in density has a major impact on the retention and selectivity of the compounds eluting in the subcritical region. However, we found that the difference in the density between the two compositions at the flow rate of 2.3 mL/min, at which the final separation was performed, was not significant (0.921 g/cm^3^ and 0.923 g/cm^3^) and the separation was influenced and improved by the supercritical phase transition to subcritical (Figure 4). The optimal flow rate was 2.3 mL per minute. Even though a higher flow rate resulted in a shortened time of analysis, it caused co-elution of the retinol (peak 10) and γ‑tocopherol (peak 11), with a resolution of 0.65 (Figure 3).

### 3.6. Hemp Seed Oil and Waste Fish Oil Analysis

**Figure 5:**
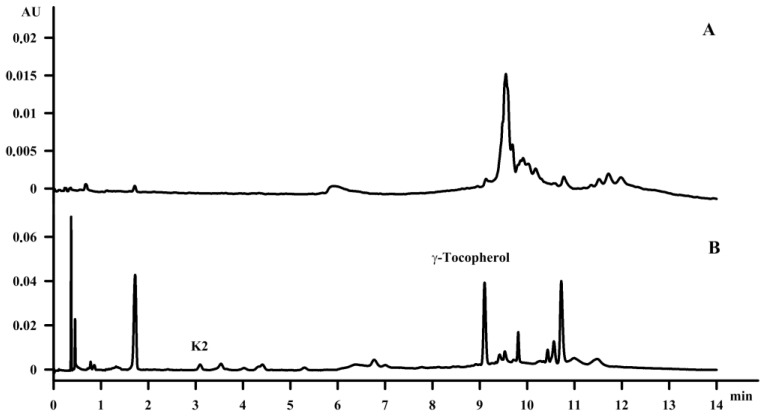
The chromatogram of hemp seed oil—before purification (**A**), after purification (**B**).

Fat-soluble vitamins were identified, separated and quantitated using the SFC method in hemp seed oil and waste fish oil; in the case of the waste fish oil this was carried out for the first time. In the analytical study of Oomah et al. [31], the analysis of tocopherols—including α-tocopherol, β-tocopherol, γ‑tocopherol and δ-tocopherol—using high-performance liquid chromatography equipped with fluorescence detection on a Primesphere (150 × 4.6 mm; 5 μm) column as well as a mixture of hexane, isopropanol and dimethyl propane as the mobile phase confirmed the presence of all tocopherols in the amounts of 3.4 mg, 0.6 mg, 73.3 mg and 2.5 mg per 100 g of oil, respectively, for the α-, β-, γ- and δ-isomers. Callaway [32] previously confirmed the presence of α‑tocopherol and γ‑tocopherol in the amount of 5 mg and 85 mg per 100 g of oil, respectively. Our study confirmed the presence of γ‑tocopherol in the amount of 10 mg per 100 g oil; additionally menaquinone-4 (K_2_-MK4) was identified in the amount of 0.4 mg per 100 g oil, which is in agreement with previous reports by Mahdinia [33] and Dam [34]. The amount of γ‑tocopherol was seven times lower than it was suggested by Callaway [32]. However, the applied saponification procedure resulted in a satisfactory concentration of the compounds in the amount of 91 mg and 1349 mg per 100 g oil for menaquinone-4 (K_2_-MK4) and γ‑tocopherol (Figure 5).

**Figure 6:**
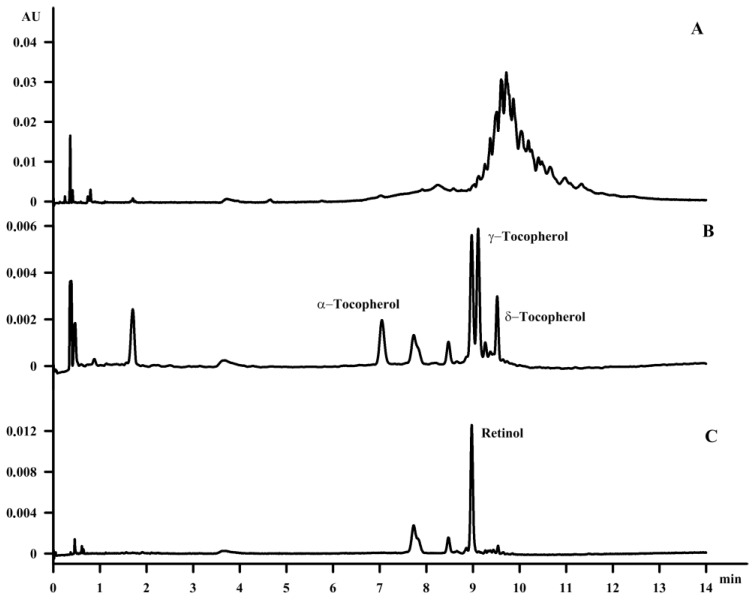
The chromatogram of fish oil, before purification (**A**), after purification (**B**-220 nm), after purification (**C**-320 nm).

Due to the lack of information on the content of vitamins in waste fish oil, it was chosen as the second material for the study. In previous studies, the content of fat-soluble vitamins was determined only in oils from different fish species [7]. However, waste fish oil is still a valuable material not only in terms of fatty acids, such as n-3, but also in terms of the fat-soluble vitamins that are present in such oil, as found in this paper. Furthermore, the content of fat-soluble vitamins constitutes the minor part of the waste fish oil, which presents some difficulties regarding identification. This is why, similarly to the case of hemp seed oil, the same saponification procedure was applied to waste fish oil and was found to be an excellent method for both the identification and purification of fat-soluble vitamins. The identified compounds were α‑tocopherol, γ‑tocopherol, δ-tocopherol and retinol, with a content of 0.3% (wt %), 2.4% (wt %), 0.27% (wt %) and 1.4% (wt %) of total waste fish oil after purification, respectively (Figure 6).

The standard addition method was successfully validated and applied to the detection and quantification of the fat-soluble vitamins in hemp seed oil and waste fish oil. The results of the oil analyses before and after purification are presented in Table 4.

## 4. Conclusions

Supercritical fluid chromatography (SFC) equipped with diode array detection allows the baseline separation of vitamin A, D, E and K. The optimized method allowed the easy separation of vitamins D and K, which are considered to be problematic when using conventional LC modes due to their similar chemical structures. Moreover, the purification method may be used as a pre-stage for the further separation of vitamins from oils with the use of, for instance, centrifugal partition chromatography. Most importantly, the method of purification is considerably low cost, and may also be an appropriate method for the identification of vitamins from complex matrices, such as the studied oils as well as, for instance, extracts obtained using supercritical fluid extraction. The experiments are to be performed in further studies.

## Figures and Tables

**Table 1:** Typical calibration data for the standard addition method for hemp seed oil and waste fish oil.

	HEMP SEED OIL					
	Before saponification			After saponification		
	Slope	Intercept	R^2^	Slope	Intercept	R^2^
menaquinone-4 (K_2_-MK4)	697.53	199.6	0.996	652.46	794.4	0.996
γ-tocopherol	200.73	1639.6	0.994	269.78	123015.3	0.996
	**WASTE FISH OIL**					
	Before saponification			After saponification		
	Slope	Intercept	R^2^	Slope	Intercept	R^2^
α-tocopherol	215.6	1140.0	0.993	512.48	63,865.17	0.993
γ-tocopherol	218.98	1422.8	0.997	104.78	10,2604.17	0.997
δ-tocopherol	210.42	580.6	0.996	362.80	40,682.33	0.993
retinol	173.42	81.4	0.996	82.04	47,256.00	0.994

Slope and intercept were obtained from the Excel slope formula and intercept formula on the basis of the obtained calibration curves (standard addition method) for analyzed compounds.

**Table 2:** Calibration data for the external calibration method for analyzing fat-soluble vitamins with the use of the UPC^2^ (ultra performance convergence chromatography) system.

Compound	Linearity Range [μg/mL]	R^2^	LOD [μg/mL]	LOQ [μg/mL]
*cis*-retinyl acetate *	5–20	0.997	0.34	0.67
*trans*-retinyl acetate	95–475	0.998	0.10	0.50
*cis*-retinyl palmitate *	5–20	0.998	0.39	0.59
*trans*-retinyl palmitate	95–475	0.999	0.45	0.87
*cis*-phylloquinone * (*cis*-K1)	5–20	0.996	0.41	0.63
phylloquinone (*trans*-K1)	95–475	0.998	0.31	0.98
menaquinone-4 (K2-MK4)	100–500	0.998	0.49	2.05
α-tocopherol	100–500	0.999	1.17	2.39
β-tocopherol	100–500	0.998	0.51	1.09
retinol	100–500	0.999	0.22	0.62
γ-tocopherol	100–500	0.999	0.43	0.89
δ-tocopherol	100–500	0.998	0.49	1.11
Cholecalciferol (D3)	100–500	0.999	0.31	1.12
Ergocalciferol (D2)	100–500	0.997	0.39	1.32

* *cis*-forms account 5% of the total standard concentration.

**Table 3:** Repeatability and intermediate precision for analyzing fat-soluble vitamins using the UPC^2^ system.

Compound	Concentration (μg/mL)	RSD (%) (*n* = 3)		Accuracy Recovery (%)	
		Repeatability	Intermediate Precision	Concentration ([µg/mL)	Mean (%) (*n* = 3)
*cis*-retinyl acetate	10	2.68	4.64	5	86.4
	15	2.14	3.24	10	86.7
	20	1.46	1.66	15	85.6
*trans*-retinyl acetate	180	2.46	4.14	95	93.7
	270	2.01	3.29	190	92.4
	360	1.56	2.04	285	94.6
*cis*-retinyl palmitate	10	2.74	4.87	5	85.9
	15	2.37	3.43	10	86.7
	20	1.77	2.15	15	87.3
*trans*-retinyl palmitate	180	2.36	4.41	95	89.7
	270	1.99	3.36	190	90.3
	360	1.46	2.55	285	91.5
retinol	75	2.73	3.99	100	94.1
	100	1.98	3.04	200	94.9
	200	1.54	2.12	300	95.3
phylloquinone (*cis*-K_1_)	10	2.87	4.88	5	86.1
	15	2.34	3.69	10	87.2
	20	1.96	2.47	15	85.3
phylloquinone (*trans*-K_1_)	180	2.79	4.32	95	91.4
	270	2.16	3.46	190	90.7
	360	1.47	2.19	285	92.9
menaquinone-4 (K_2_-MK4)	75	2.54	4.65	100	97.2
	100	2.02	3.14	200	96.1
	200	1.73	2.17	300	97.3
α-tocopherol	75	2.67	4.39	100	87.3
	100	2.21	3.10	200	86.9
	200	1.65	1.97	300	91.7
β-tocopherol	75	2.21	4.74	100	92.8
	100	1.98	3.99	200	92.1
	200	1.44	2.21	300	93.7
γ-tocopherol	75	2.54	4.24	100	110.9
	100	2.12	3.95	200	112.4
	200	1.79	2.39	300	112.9
δ-tocopherol	75	2.87	4.30	100	93.7
	100	2.33	3.47	200	91.3
	200	2.09	2.65	300	89.4
cholecalciferol (D_3_)	75	2.41	5.41	100	86.3
	100	1.27	2.64	200	89.4
	200	1.10	1.54	300	91.8
ergocalciferol (D_2_)	75	2.46	4.12	100	84.7
	100	1.87	3.15	200	88.7
	200	1.12	2.71	300	93.8

**Table 4:** Content of fat-soluble vitamins in the hemp seed oil and waste fish oil before and after the saponification procedure.

Fat-Soluble Vitamin	HEMP SEED OIL		WASTE FISH OIL	
	Before Purification	After Purification	Before Purification	After Purification
	Mean (Range) (mg/g) (*n* = 3)			
*trans*-retinyl acetate	−	−	−	−
*trans*-K_1_	−	−	−	−
menaquinone (K_2_-MK4)	0.004	0.006	−	−
*trans*-retinyl palmitate	−	−	−	−
α-tocopherol	−	−	1.287	1.519
retinol	−	−	0.069	0.703
β-tocopherol	−	−	−	−
γ-tocopherol	0.1	0.135	1.585	1.194
δ-tocopherol	−	−	1.345	1.367
cholecalciferol (D_3_)	−	−	−	−
ergocalciferol (D_2_)	−	−	−	−

− Meaning not identified.
